# Effects of Green Lettuce Leaf Extract on Sleep Disturbance Control in Oxidative Stress-Induced Invertebrate and Vertebrate Models

**DOI:** 10.3390/antiox10060970

**Published:** 2021-06-17

**Authors:** Kyungae Jo, Singeun Kim, Yejin Ahn, Hyung Joo Suh

**Affiliations:** 1 Department of Integrated Biomedical and Life Science, Graduate School, Korea University, Seoul 02841, Korea; kyungae11@korea.ac.kr (K.J.); kimsingun@gmail.com (S.K.); cassandra7@hanmail.net (Y.A.); 2 Transdisciplinary Major in Learning Health Systems, Department of Healthcare Sciences, Graduate School, Korea University, Seoul 02841, Korea

**Keywords:** green lettuce leaf extract, sleep, oxidative stress, GABA receptor, *Drosophila melanogaster*, rodents

## Abstract

This study investigated the effect of ethanol-extracted green lettuce leaf (GLE) on sleep behavior in physical stress-induced invertebrate and vertebrate models. In *Drosophila melanogaster*, the group that experienced vibration stress showed decreased sleep time compared to the no-vibration-stress control group, but the GLE treatment group recovered this lost sleep time. The GLE group also recovered the gene expression of downregulated superoxide dismutase induced by vibration stress conditions. According to electroencephalography analysis of rats, non-rapid eye movement (NREM) sleep significantly decreased with a decrease in sleep time for the group in which immobilization stress was induced. In the GLE group (120 mg/kg), the change in sleep pattern caused by stress was restored, and NREM sleep increased by 68.8%, improving overall sleep quality. In addition, GLE upregulated the expression levels of oxidation-related factors and γ-aminobutyric acid (GABA_A_) receptor. Quercetin-3-glucuronide (Q3G) was evaluated as a sleep-promoting active substance contained in GLE using the pentobarbital-induced sleep test and showed the effect of prolonged sleep time. Q3G inhibited [^3^H]-flumazenil binding in a concentration-dependent manner with GLE. Taken together, the results indicate that GLE effectively binds to the GABA_A_ receptor to promote sleep, demonstrating the potential of Q3G as an active substance.

## 1. Introduction

Sleep is an important health issue because not only is sleep of a sufficient quality and amount necessary for humans to perform normal activities, but also sleep deprivation leads to fatigue, aggression, and anxiety [1]. Without adequate sleep, humans experience a loss of energy and vitality, and a variety of health problems arise. According to Atrroz and Salim [2], reactive oxygen species (ROS) that accumulate during waking periods are removed during sleep. While awake, a large amount of oxygen in the brain is consumed by metabolism, as occurs during electron transfer, which increases ROS levels. During sleep, antioxidant activity is enhanced to protect the brain from ROS. It has also been reported that ROS is associated with sleep-deprivation processes [2,3]. Sleep reduces oxidative stress and is involved in recovery [4].

Several studies have reported that sleep plays a role in removing ROS that has accumulated in the brain, but there are also reports that contradict these results. Sleep deprivation has been shown to decrease the ability to eliminate oxidative stress in the brains of rats and mice [5,6]. In contrast, another study reported that there was no change in the ability to remove oxidative stress due to lack of sleep [7]. However, similar to the effects of melatonin, it has been reported that controlling sleep or enhancing antioxidant activity helps to recover various physiological changes caused by sleep deprivation [8].

*Lactuca sativa* has traditionally been used to treat anxiety, insomnia, and neurosis; the leaves in particular are known to promote sedative and hypnotic effects [9,10]. Lactucin and lactucopicrin are known to be contained in lettuce as a hypnotic substance that induces sleep and gives a bitter taste [11]. In addition, it contains various polyphenols such as caftaric acid, chlorogenic acid, chicoric acid, gallic acid, catechin, ellagic acid, rutin, quercetin and scutellarin [12].

In previous studies, the sleep-enhancing activity of green romaine lettuce, a type of *Lactuca sativa*, was confirmed in a rat model [13]. In the pentobarbital-induced sleep test, romaine lettuce leaf extract reduced sleep latency and increased sleep time. This extract also increased non-rapid eye movement (NREM) sleep in a Sprague–Dawley (SD) rat model, while reducing rapid eye movement (REM) sleep, as assessed by electroencephalography (EEG). By examining the antagonistic behavior of sleep-related receptors, it was confirmed that the sleep-enhancing activity of green romaine lettuce extract was due to the GABAergic mechanism.

However, studies on the effect of another type of *Lactuca sativa*, green lettuce, on sleep enhancement and its correlation with oxidative stress have not been conducted in invertebrate or vertebrate models. In this study, we evaluated the correlation between sleep and oxidative stress in stress-induced invertebrate and vertebrate models and confirmed the effects of green lettuce leaf extract (GLE) administration. In addition, the basis of GLE-mediated sleep promotion was investigated through competitive binding activity analysis to identify the active substances of GLE and analyze their mechanism of action.

## 2. Materials and Methods

### 2.1. Plant Material and Preparation of Extracts

Dried green lettuce leaves (100 g) were extracted with 500 mL of 70% ethanol for 2 h at 70 °C. After this process was repeated three times, the combined extracts were filtered, concentrated with a rotary evaporator (R-100, BUCHI Labortechnik AG, Flawil, Switzerland), and freeze-dried (Ilshin Biobase Co., Gyeonggi, Korea).

### 2.2. Drosophila Melanogaster Stocks

*Drosophila* Canton-S strain was obtained from the Bloomington Stock Center (Indiana University, Bloomington, IN, USA) and maintained in a 12 h/12 h light–dark cycle on media (cornmeal, dried yeast, sucrose, agar, propionic acid, and p-hydroxybenzoic acid methyl ester) in incubators (25 °C, 60% relative humidity). Three-day-old male flies were collected under CO_2_ anesthesia for analysis [14]. To evaluate the locomotor activity and survival rate of *Drosophila* treated with green lettuce leaf extract (GLE, 0.5% and 1.0%), each concentration was added to 5% sucrose and 2% agar media, and *Drosophila* were exposed for nine days. Meanwhile, *Drosophila* were exposed to media containing GLE for 14 days to assess gene expression levels.

### 2.3. Vibration Stress in Drosophila Melanogaster

Vibration stress was induced by modifying the methods of a previous study [10]. Ten *Drosophila* were maintained in each vial (25 mm width and 100 mm length) and placed in a vibration device (Brὓel & Kjoer, type 4810) or normal conditions in an incubator. Vibration of 300 Hz was applied for 20 s, followed by a 10 s pause. This was repeated for 15 min, followed by a 30 min break. This cycle was applied 12 times a day (total 9 h), and stress was applied through vibration for six out of nine days.

### 2.4. Behavioral Tests in Drosophila Melanogaster

To analyze total activity, a locomotor activity monitoring (LAM) system (TriKinetics, Waltham, MA, USA) was used to evaluate the sleep-promoting effects of GLE. Ten *Drosophila* were placed in a vial and acclimated for three days in constant darkness. After adaptation, vibration stress was applied for two days, and behavior patterns were analyzed for one day. Actogram J was used for data analysis, and sleep parameters were calculated based on the total number of activities recorded each day [15].

### 2.5. Animals

Male Sprague–Dawley rats and ICR mice (six weeks old) were purchased from Orient Bio (Orient Bio Inc., Seongnam, Korea). All animals were housed in cages at 24 ± 2 °C and 55% relative humidity with a 12 h/12 h light–dark cycle. Food and water were freely available and acclimatized to the vivarium for at least one week to perform the test. Afterwards, rats (*n* = 8/group) and mice (*n* = 7/group) were randomly grouped. All animal experiments were approved by the Korea University Institutional Animal Care and Use Committee (KUIACUC-2019-20, Seoul, Korea).

### 2.6. Immobilization Stress Procedure in Rats

Rats were stressed for 7 h (10.00 to 17.00 h) daily for 14 days in an immobilization cage (8 cm width, 12 cm length, 12 cm height). Animals in the no-immobilization-stress control group were not subjected to any type of stress and were kept separately in standard cages [16]. GLE was administered for nine days after induction of immobilization stress. At 9 am, 80 and 120 mg/kg concentrations of GLE were orally administered to the two treatment groups, and saline was orally administered to the control groups.

### 2.7. EEG Recordings and Analysis in Rats

Electrode insertion surgery was performed one week before induction of immobilization stress. Electroencephalography (EEG) analysis was performed 24 h a day for nine days of oral administration of GLE. Electrode implantation surgery and EEG analysis were performed as described previously [17].

### 2.8. Measurement of Hydrogen Peroxide (H_2_O_2_) and Malondialdehyde (MDA)

Concentrations of hydrogen peroxide (H_2_O_2_) and malondialdehyde in the rat brain were analyzed after nine days of treatment with GLE (80 or 120 mg/kg). The measurement of H_2_O_2_ content from oxidative stress was conducted using an Oxi Tec assay kit (BIOMAX, Rockville, MD) at Ex/Em 540/590 nm. The MDA assay was conducted following the method of Buege and Aust [18], with some minor modifications. Tissues were homogenized with Tris-HCl buffer (0.4 M; pH 7.4), centrifuged for 10 min at 4000× *g*, and the supernatant was added to a reaction buffer (8.1% sodium dodecyl sulfate, 0.8% TBA, 20% acetic acid). The mixture was vortexed and incubated at 100 °C for 60 min. After cooling, butanol was added to the mixture and centrifuged at 8000× *g* for 5 min. The supernatant was collected, and the absorbance was measured at 532 nm. The content of each component was expressed based on the amount of protein in the sample.

### 2.9. mRNA Expression of Oxidative-Related Factor and Neurotransmitter Receptors

GLE was administered to *Drosophila* for two weeks, and GLE was orally administered for nine days in rats. Heads and brains, respectively, were obtained, and total RNA was isolated using TRIzol reagent (Invitrogen, CA, USA). mRNA expression was analyzed by quantitative real-time RT-PCR using the StepOne Plus Real-time PCR system (Applied Biosystems, CA, USA) with a Power TaqMan PCR Master Mix Kit (Applied Biosystems), as previously described [17]. The following genes were analyzed by qRT-PCR: SOD (NM_057387.5), SOD2 (NM_001299574.1), CAT (NM_080483.3), GstD1 (NM_001038953.2), Rdl (NM_001274688.1), GABA_B_-R1 (NM_001259104.2), GABA_B_-R2 (NM_001300527.1), 5HT (NM_166322.2), and RpL32 (NM_001144655.3) in the *Drosophila* model; SOD (NM_017050.1), SOD2 (NM_017051.2), CAT (NM_012520.2), GPx1 (NM_030826.3), α_2_-GABA_A_ (NM_017289.1), GABA_B_-R1 (NM_031028.3), GABA_B_-R2 (NM_001081141.1), 5-HT1A (NM_012585.1), and β-actin (NM_031144.4) in the rat model.

### 2.10. Analysis of Polyphenols Contained in GLE

Polyphenol analysis was carried out on an HPLC system (Waters Scientific Ltd., Mississauga, ON, Canada) equipped with a Meteoric Core C18 column (S-2.7 μm, 8 nm, 150 mm × 4.6 mm, Agilent, Santa Clara, CA 95051, USA). The mobile phase was analyzed under gradient elution conditions using 0.5% formic acid in water (A) and 0.5% formic acid in acetonitrile (B). The flow rate of the mobile phase was 0.3 mL/min, and the sample injection volume was 5 μL, which was detected at 280 nm.

### 2.11. Pentobarbital-Induced Sleep Test

Mice were fasted for more than 12 h before the experiment, which was performed between 14.00 to 17.00 h. GLE (80, 120 mg/kg) or chlorogenic acid, scutellarin, and Q3G (5, 10, 20 mg/kg) was administered orally, and pentobarbital (42 mg/kg) was then intraperitoneally injected 45 min later. After injection, mice were moved to individual cages, and sleep latency and duration were measured. Sleep latency time is the time from pentobarbital injection to sleep onset, and sleep duration time is the time between right reflex loss and recovery [19].

### 2.12. GABA_A_-BDZ Receptor-Binding Assay

A GABA_A_-benzodiazepine (BDZ) receptor-binding assay was carried out by isolating receptors from the rat cerebral cortex as previously described [17]. The displacement of [^3^H]-flumazenil binding activity was measured by administering GLE, chlorogenic acid, scutellarin, and Q3G at concentrations of 0.1, 1, and 10 mg/mL each.

### 2.13. Statistical Analysis

Significant differences between groups were evaluated by one-way analysis of variance (ANOVA) and Tukey’s multiple range test using statistical package for the social sciences (SPSS) 12.0 version for Windows (SPSS Inc., Chicago, IL, USA), and a *p*-value of less than 0.05 was considered significant. All data are expressed as the mean ± standard error of the mean (SEM).

## 3. Results

### 3.1. Effects of Green Lettuce Leaf Extract (GLE) on Locomotor Activity in Vibration-Stressed Drosophila Melanogaster

**Figure 1:**
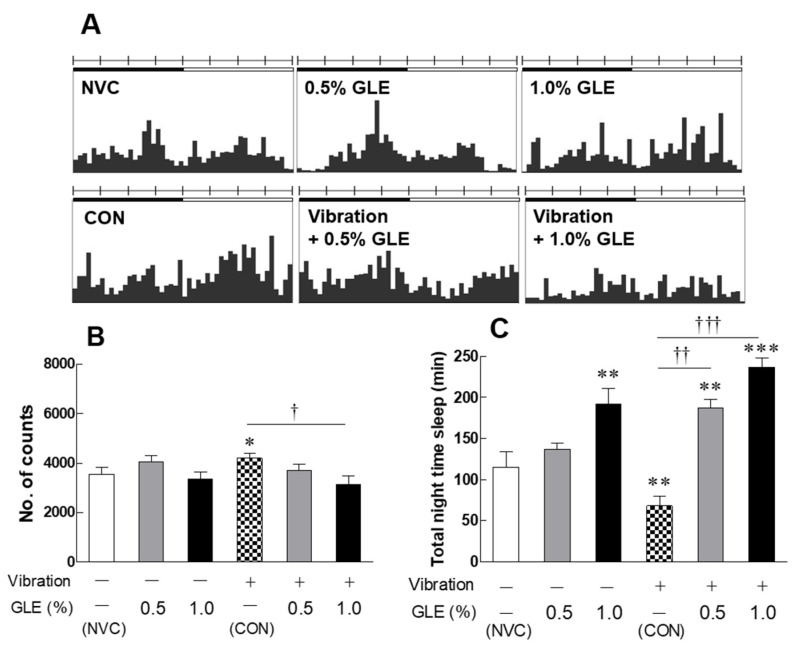
Effects of GLE (green lettuce leaf extract) on behaviors changed by vibration stress in fruit flies. (**A**) Representative actograms for the locomotor activity. The bar above each actogram indicates night (in black) and day (in white) h. (**B**) Nighttime activity and (**C**) duration of nighttime sleep in the locomotor activity monitoring (LAM) system. Experimental groups include the no-vibration-stress control group (NVC), control group (CON, stress-induced vibration), and GLE (green lettuce leaf extract)-treated groups with or without stress (0.5 and 1.0%). Values are presented as the mean ± standard error of the mean (SEM) for each group, *n* = 150. * *p* < 0.05, ** *p* < 0.01, and *** *p* < 0.001 when compared with the NVC group. † *p* < 0.05, †† *p* < 0.01 and ††† *p* < 0.001 when compared with the stress CON group (ANOVA followed by post-hoc Tukey’s test).

Actograms showed that GLE effectively regulates the circadian rhythm of altered Drosophila under stress conditions, in both the daytime (white bar) and nighttime (black bar) (Figure 1A). The nighttime activity of Drosophila exposed to stress (CON; control group) increased significantly compared to the no-vibration-stress control (NVC) group (Figure 1B, *p* = 0.0382), and the sleep time tended to decrease (Figure 1C, *p* = 0.0062). The group treated with only GLE, without stress, showed no significant difference in activity during the nighttime compared to the NVC group, but the high-concentration GLE (1.0%) group showed significantly increased sleep time (*p* = 0.0047). On the other hand, in the group treated with GLE with stress (0.5, 1.0%), the sleep time was significantly increased in a dose-dependent manner, and in particular, the high-concentration GLE group showed a 71.3% increase in sleep time compared to the CON group (*p* = 0.0006).

After confirming the survival rate for nine days during the behavioral analysis as vibration stress was applied, the survival rate for the CON group was shown to decrease by 24% compared to the NVC group on the ninth day (Figure S1). Meanwhile, the groups treated with GLE (0.5, 1.0%) with stress recovered the reduced survival rate; the recovered rate was observed to be higher for the GLE group than for the NVC group (98.7% and 98.0%, respectively).

### 3.2. Effects of Green Lettuce Leaf Extract (GLE) on Brain Receptor Expression in Vibration-Stressed Drosophila Melanogaster

**Figure 2:**
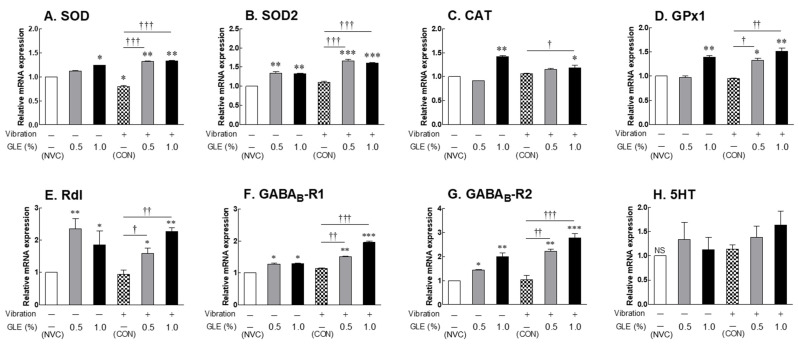
Effects of GLE (green lettuce leaf extract) on (**A**) SOD, (**B**) SOD2, (**C**) CAT, (**D**) GstD1, (**E**) Rdl, (**F**) GABAB-R1, (**G**) GABAB-R2, and (**H**) 5HT mRNA expression by vibration stress in fruit flies. Fly heads were collected after 12 h/12 h cycles for two weeks. Experimental groups include the no-vibration-stress control group (NVC), control group (CON, stress-induced vibration), and GLE (green lettuce leaf extract)-treated groups with or without stress (0.5 and 1.0%). Values are presented as the mean ± standard error of the mean (SEM) for each group, *n* = 150. * *p* < 0.05, ** *p* < 0.01, and *** *p* < 0.001 when compared with the NVC group. † *p* < 0.05, †† *p* < 0.01, and ††† *p* < 0.001 when compared with the CON group (ANOVA followed by post-hoc Tukey’s test). NS, not significant.

Analysis of the expression of oxidative stress- and sleep-related brain receptor genes revealed that the amount of superoxide dismutase (SOD) expression significantly decreased for the CON group exposed to vibrational stress compared to that in the NVC group (Figure 2A, *p* = 0.0276). However, there was no significant difference in SOD2, catalase (CAT), or glutathione S transferase D1 (GstD1) (Figure 2B–D). In contrast, the group treated with 1.0% GLE without stress significantly increased the level of expression compared to the NVC group for all SOD (*p* = 0.0234), SOD2 (*p* = 0.0061), CAT (*p* = 0.0042), and GstD1 (*p* = 0.0027). Similarly, groups treated with GLE and stress showed a statistically significant increase in the expression levels of SOD (*p* = 0.0002), SOD2 (*p* = 0.0003), CAT (*p* = 0.0324), and GstD1 (*p* = 0.0052) compared to the CON group. In particular, the other antioxidant regulators, with the exception of GstD1, showed higher expression levels in the group treated with GLE with stress than in the group treated with GLE without stress (*p* = 0.0351, *p* = 0.0065, respectively).

There was no significant difference in sleep-related brain receptors compared to the NVC group for both GABA and serotonin receptors in Drosophila exposed to vibrational stress (Figure 2E–H). On the other hand, it was confirmed that the gene expression levels of Rdl (*p* = 0.0075, *p* = 0.0412), GABA_B_-R1 (*p* = 0.0345), and GABA_B_-R2 (*p* = 0.0215, *p* = 0.0051) were significantly increased in the groups treated with only GLE without stress compared to the NVC group (Figure 2E–G). In addition, the GLE and stress-exposed groups showed a statistically significant increase in the gene expression levels of Rdl (*p* = 0.00395, *p* = 0.0039), GABA_B_-R1 (*p* = 0.0048, *p* = 0.0005), and GABA_B_-R2 (*p* = 0.0028, *p* = 0.0003) compared to the CON group, as in the GLE-only groups. In the case of 5HT, a serotonin receptor, the expression level showed a tendency to increase with GLE treatment, but there was no significant difference between the NVC and CON groups (Figure 2H).

### 3.3. Effect of Green Lettuce Leaf Extract (GLE) on Sleep Architecture Changed by Immobilization Stress in Rats

**Figure 3:**
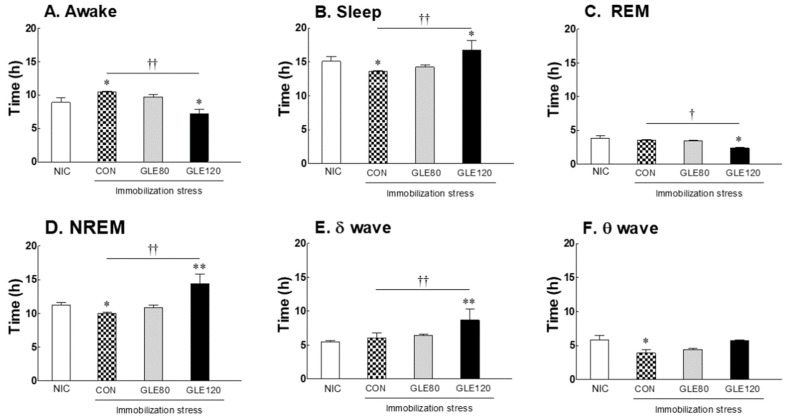
Effects of GLE (green lettuce leaf extract) on (**A**) awake, (**B**) sleep, (**C**) REM, (**D**) NREM, (**E**) δ-wave, and (**F**) θ-wave sleep pattern changed by immobilization stress in rats. EEG analyses were conducted for nine days, and GLE was administered orally. Experimental groups include the no-immobilization-stress control group (NIC), control group (CON, stress-induced immobilization), and GLE (green lettuce leaf extract)-treated groups with stress (80 and 120 mg/kg). Values are presented as the mean ± standard error of the mean (SEM) for each group, *n* = 8. * *p* < 0.05 and ** *p* < 0.01 when compared with the NIC group. † *p* < 0.05 and †† *p* < 0.01 when compared with the CON group (ANOVA followed by post-hoc Tukey’s test).

To evaluate the correlation between sleep and oxidative stress in vertebrates based on the results of the Drosophila model, SD rats were immobilized for two weeks, then EEG analysis was conducted with oral administration of green lettuce leaf extract (GLE) for nine days. The immobilization stress control (CON) group showed a 14.7% increase in awake time and a 10.1% significant decrease in sleep time compared to the no-immobilization-stress control (NIC) group (Figure 3A,B; *p* = 0.0378). In the stressed group, the decrease in sleep time was found to be potentially due to a significant decrease in NREM sleep (Figure 3D, *p* = 0.0412). Meanwhile, it was confirmed that the group treated with a high concentration (120 mg/kg) of GLE (GLE120) along with immobilization stress recovered the reduced sleep time due to stress. The GLE120 group showed a significant increase in sleep time compared to the CON group (*p* = 0.0063), which was due to the 68.8% significant increase in NREM sleep (*p* = 0.0036). In the GLE120 group, the delta wave, which is deep sleep during NREM sleep, was significantly increased compared to the CON group (Figure 3E, *p* = 0.0047), whereas REM sleep decreased (Figure 3C, *p* = 0.0324), confirming the effect of improved sleep quality when GLE was administered.

### 3.4. Effect of Green Lettuce Leaf Extract (GLE) on ROS Production by Immobilization Stress in Rats

**Figure 4:**
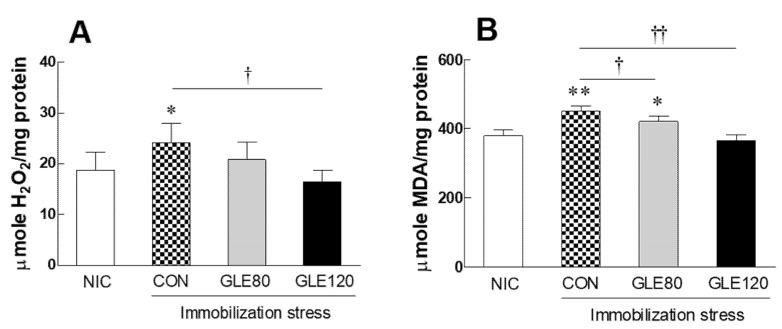
Effects of GLE (green lettuce leaf extract) on (**A**) hydrogen peroxide and (**B**) malondialdehyde by immobilization stress in rats. Experimental groups include the no-immobilization-stress control group (NIC), control group (CON, stress-induced immobilization), and GLE (green lettuce leaf extract)-treated groups with stress (80 and 120 mg/kg). Values are presented as the mean ± standard error of the mean (SEM) for each group, *n* = 8. * *p* < 0.05 and ** *p* < 0.01 when compared with the NIC group. † *p* < 0.05 and †† *p* < 0.01 when compared with the CON group (ANOVA followed by post-hoc Tukey’s test).

Reactive oxygen species (ROS) have been reported to be strongly associated with insomnia. In addition, endogenous ROS produced from aerobic metabolism may be the most important cause of nerve damage. Here, the effect of GLE administration on ROS in rat brain tissue induced by immobilization stress was confirmed. The production of hydrogen peroxide was significantly higher in the CON group than in the NIC group (*p* = 0.0312), and when treated with high concentrations of GLE (GLE120), it decreased significantly compared to the CON group (*p* = 0.0189), showing a level similar to that of the NIC group (Figure 4A). Similarly, due to stress, the amount of malondialdehyde significantly increased compared to the NIC group (*p* = 0.0076), which was significantly reduced according to the concentration of GLE treatment (Figure 4B, *p* = 0.0415 and *p* = 0.0054, respectively).

### 3.5. Effects of Green Lettuce Leaf Extract (GLE) on Brain Receptor Expression by Immobilization Stress in Rats

**Figure 5:**
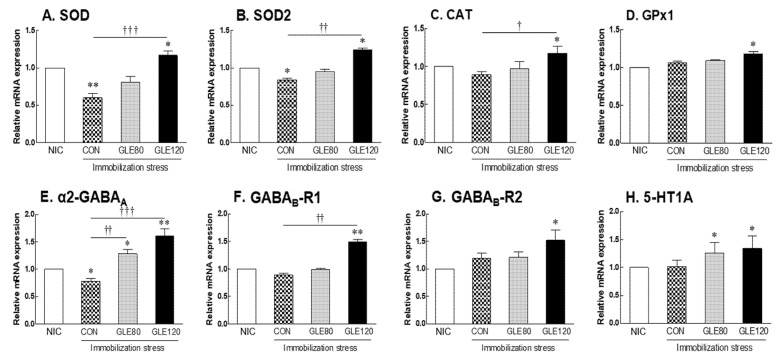
Effects of GLE (green lettuce leaf extract) on (**A**) SOD, (**B**) SOD2, (**C**) CAT, (**D**) GPx1, (**E**) α_2_-GABA_A_, (**F**) GABA_B_-R1, (**G**) GABA_B_-R2, and (**H**) 5-HT1A mRNA expression by immobilization stress in rats. Brain tissue was collected nine days after oral administration. Experimental groups include the no-immobilization-stress control group (NIC), control group (CON, stress-induced immobilization), and GLE (green lettuce leaf extract)-treated groups with stress (80 and 120 mg/kg). Values are presented as the mean ± standard error of the mean (SEM) for each group, *n* = 8. * *p* < 0.05 and ** *p* < 0.01 when compared with the NIC group. † *p* < 0.05, †† *p* < 0.01, and ††† *p* < 0.001 when compared with the CON group (ANOVA followed by post-hoc Tukey’s test). α_2_-GABA_A_: GABA_A_ receptor subunit alpha2.

The gene expression of antioxidant regulators in a rat brain during immobilization stress was analyzed, and the levels of SOD and SOD2 genes in the CON group exposed to stress were significantly decreased 1.67-fold and 1.19-fold, respectively, compared to the NIC group (Figure 5A,B; *p* = 0.0053 and *p* = 0.0175, respectively). On the other hand, in the group treated with high concentrations of GLE (GLE120), SOD and SOD2, which were reduced by stress, were recovered, showing a significant 1.95-fold and 1.48-fold increase, respectively, compared to the CON group (*p* = 0.0003 and *p* = 0.0021, respectively). In addition, the GLE120 group showed a significantly increased expression of CAT and GPx1 compared to the NIC group (Figure 5C,D; *p* = 0.0312 and *p* = 0.0432, respectively).

Additionally, sleep-related GABA and serotonin receptor expression levels were analyzed. In the immobilization stress group, the α_2_-GABA_A_ expression level significantly decreased 0.78-fold compared to that in the NIC group (Figure 5E, *p* = 0.0275). However, according to the concentration of GLE administered, the α_2_-GABA_A_ expression level, compared to the CON group, increased 1.64-fold and 2.06-fold, respectively (*p* = 0.0031, *p* = 0.0003). Similarly, GABA_B_-R1, GABA_B_-R2, and 5-HT1A showed a tendency to increase significantly in the GLE120 group, compared to the NIC group (Figure 5F–H, *p* = 0.0026, *p* = 0.0371, and *p* = 0.0418), but there was no change due to immobilization stress induction.

### 3.6. Analysis of Green Lettuce Leaf Extract (GLE)

**Figure 6:**
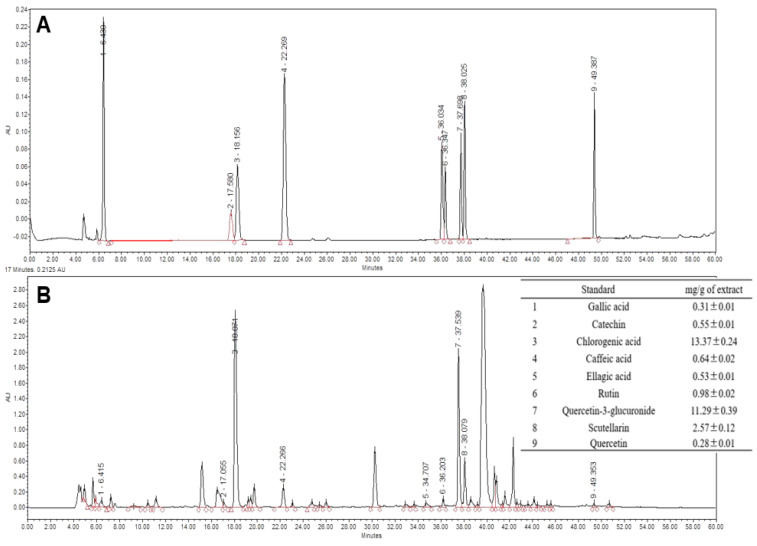
HPLC chromatogram of (**A**) standard references comprised of polyphenols and (**B**) green lettuce leaf extract.

The sesquiterpene lactones, such as lactucin and lactucopicrin, which are commonly known sleep-promoting substances in lettuce, were analyzed using an HPLC system. Lactucin and lactucopicrin were present in small amounts—3.76 and 3.90 µg/g of extract, respectively—so they are not suitable as sleep-active substances (data not shown). Therefore, the contents of chlorogenic acid, Q3G, scutellarin, rutin, caffeic acid, catechin, ellagic acid, gallic acid, and quercetin in GLE were analyzed by HPLC (Figure 6). Among them, the contents of chlorogenic acid, Q3G, and scutellarin, which are known to have a sleep or sedative effect, were relatively high at 13.37 ± 0.24, 11.29 ± 0.39 and 2.57 ± 0.12 mg/g of extract, respectively.

### 3.7. Effects of Green Lettuce Leaf Extract (GLE), Chlorogenic Acid, Scutellarin, and Q3G-Mediated Sleep Behavior in a Pentobarbital-Induced Sleep Mouse Model

**Figure 7:**
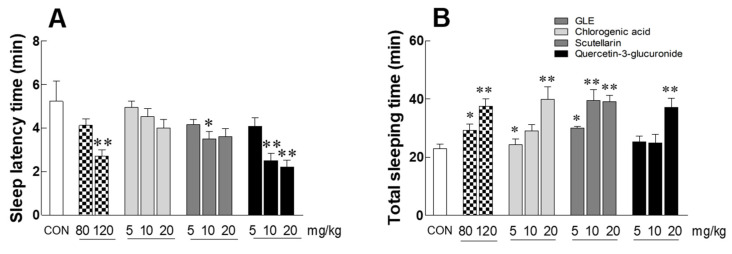
Effects of GLE (green lettuce leaf extract), chlorogenic acid, scutellarin, and quercetin-3-glucuronide (Q3G) on (**A**) sleep latency time and (**B**) total sleep time in mice that received a hypnotic dose of pentobarbital (42 mg/kg, i.p.). Experimental groups include the control group (CON), GLE (green lettuce leaf extract)-treated groups (80 and 120 mg/kg), chlorogenic acid, scutellarin, and Q3G-treated groups (5, 10, and 20 mg/kg). Values are presented as the mean ± standard error of the mean (SEM) for each group, *n* = 7. * *p* < 0.05 and ** *p* < 0.01 when compared with the CON group (ANOVA followed by post-hoc Tukey’s test).

Through a pentobarbital-induced sleep experiment in mice, we evaluated the sleep-enhancing effects of chlorogenic acid, scutellarin, and Q3G, the expected sleep-active substances of GLE (Figure 7). At the hypnotic dose of pentobarbital (42 mg/kg, i.p.), scutellarin (10 mg/kg) and Q3G (10 and 20 mg/kg) significantly reduced the sleep latency time compared to the CON group (Figure 7A, *p* = 0.0412, *p* = 0.0062, and *p* = 0.0041, respectively). In particular, the sleep latency time at 10 mg/kg of Q3G was 2.5 ± 0.35 min, which was similar to that of the 120 mg/kg GLE treatment group (2.71 ± 0.29 min). In addition, the total sleeping time increased significantly in groups with high concentrations (20 mg/kg) of chlorogenic acid, scutellarin, and Q3G compared to the CON group (Figure 7B, *p* = 0.0051), and the GLE-administered group showed a significant increase in sleep time in a concentration-dependent manner (80 mg/kg: 29.25 ± 2.12 min, 120 mg/kg: 37.52 ± 2.62 min, respectively).

### 3.8. GABA_A_-BDZ Receptor-Binding Activity of Green Lettuce Leaf Extract (GLE), Chlorogenic Acid, Scutellarin, and Q3G

The binding activity of GLE and expected sleep-active substances to GABA_A_-benzodiazepine (BDZ) receptors was determined using the radioligand [^3^H]-flumazenil. Table 1 summarizes the sample-mediated displacement of [^3^H]-flumazenil binding lrb% found in the present study. GLE increased the displacement of [^3^H]-flumazenil from 1.36 to 16.17% in a concentration-dependent manner. In contrast, chlorogenic acid and scutellarin did not show binding activity to the GABA_A_-BDZ receptor. The concentration of Q3G was increased from 0.1 to 10 mg/mL, and binding increased significantly from 16.58% to 88.13%. In particular, at a concentration of 10 mg/mL, it was confirmed that the Q3G group increased in binding activity by about 5.45 times compared to the GLE group. These results show that GLE effectively binds to the GABA_A_ receptor, and Q3G, which is contained in GLE, is the main substance that exhibits sleep activity and promotes sleep.

## 4. Discussion

Currently, medicines used for the treatment of sleep disorders or insomnia adversely affect quality of life due to their various side effects [20]. Most medicines prescribed for insomnia carry the risk of overdose, tolerance, habituation, and addiction. Natural sleep aids are widely used as alternatives to prescription medicines to improve sleep quality and to avoid side effects, including impaired cognitive function, tolerance, and addiction disorders [21]. The demand for sleep-related dietary supplements derived from natural sources has increased dramatically in recent years owing to their safety and minimal negative side effects. Many studies have reported that natural products alleviate sleep disorders. *Polygonatum sibiricum*, *Nelumbo nucifera* extracts, and a mixture of *Valeriana officinalis* and hops have been reported to increase sleep time in animal models [17,22,23]. In addition, *Lactuca sativa* extract improves sleep quality and increases total sleep time [13]. However, previous studies have not investigated the effect of green lettuce leaf extract on sleep enhancement and its correlation with oxidative stress in invertebrate or vertebrate models.

In this study, we used a *Drosophila* model that induces oxidative stress due to vibration and exposed green lettuce leaf extract (GLE) to the media to assess sleep-promoting activity. Sleep time was significantly reduced in night activities due to vibration stress, but the GLE treatment group with vibration recovered the reduced sleep time. In addition, the group treated with GLE with vibration showed higher sleep time than the group treated with only GLE without vibration stress (Figure 1). By analyzing genes related to oxidation in the *Drosophila* head exposed to vibrational stress, it was confirmed that oxidative stress was induced by vibration due to decreased SOD expression. On the other hand, in the group treated with GLE with vibration stress, SOD, SOD2, CAT, and GstD1 were significantly increased (Figure 2A–D). According to a previous study, the expression of antioxidant-related genes is reduced by chemical stress, and the gene expression of SOD, SOD2, CAT, and GstD1 in the group treated with *Sanguisorba officinalis* and *Zedoariae rhizoma* extracts in *Drosophila* was restored to normal levels [24]. In addition, in *Drosophila* exposed to vibrational stress, there was no change in the expression level of genes related to sleep, but the expression of GABA_A1_ (Rdl), GABA_B_-R1, and GABA_B_-R2 was increased during GLE treatment (Figure 2E–H).

Based on previous studies in which sleep patterns changed due to immobilization stress in rodents [16,25,26], we confirmed the sleep-promoting effect and mechanism of action of GLE. As shown in Figure 3, rats with immobilization stress decreased their sleep time, while they recovered to a significant level after GLE administration. It was confirmed that immobilization stress causes a decrease in NREM sleep, and GLE improves the quality of sleep by improving the duration of delta waves during NREM sleep. The sleep cycle has two patterns—NREM and REM sleep—both of which are controlled by the autonomic nervous system [27]. NREM sleep, a slow-wave form of sleep, is known to induce relaxed- and deep-sleep patterns. In contrast, REM sleep is characterized by active brain waves, with its intensity caused by a gradual increase in parasympathetic activity and a decrease in sympathetic activity [28]. The relative levels of these two types of sleep are related to an individual’s quality of sleep. In particular, tissue and immune system restoration occurs during NREM sleep [29]. In the present study, we found that NREM sleep was increased while REM sleep was decreased in GLE-administered animals, a pattern that indicates that GLE improves sleep quality as well as sleep duration.

Our results also showed that rat brain tissue treated with immobilization stress increased H_2_O_2_ and MDA levels compared to the NIC group. In the rat model, GLE intake decreased H_2_O_2_ production, and the amount of MDA, a lipid peroxidation product, decreased with increasing GLE concentration (Figure 4). In particular, similar to the results in the invertebrate model, the expression of the SOD and SOD2 genes in the immobilization stress group was decreased, and the GLE-mediated increase in antioxidant enzyme activity was found to be due to the upregulation of the gene encoding the enzyme (Figure 5A–D). Enzymatic antioxidant defenses include SOD, CAT, and GPx [2], which reduce SOD activity in the hippocampus and brainstem of rats with chronic sleep deprivation [5]. In many studies, flavonoids and phenolic compounds distributed in large amounts in plants have been reported to exhibit antioxidant properties [30,31,32,33]. Sesquiterpene lactones, lactucin, deoxylactucin, lactucopicrin, and guaianolide, which are present in lettuce extracts, can prevent lipid peroxidation due to their antioxidant activity [34,35,36].

Furthermore, α_2_-GABA_A_, downregulated by immobilization stress, recovered to a higher level than in the no-immobilization-stress control group with GLE treatment, and brain receptors, such as GABA_B_ and 5-HT1A, were increased in the group treated with high concentrations of GLE (Figure 5E–H). These compounds are known to contain substances that induce sleep; the main ingredients are lactucin, lactucopicrin, and their derivatives, which are sesquiterpene lactones, substances that give a bitter taste and have been reported as active substances [11,37]. In a previous study, we evaluated the sleep-enhancing effect of green romaine lettuce in the rodent model, and the contents of lactucin and lactucopicrin analyzed by HPLC were 1071.1 and 199.2 μg/g of extract, respectively. It has been shown that the lactucin and lactucopicrin contained in romaine lettuce promote sleep through a GABAergic mechanism [13]. On the other hand, as a result of analyzing the contents of lactucin and lactucopicrin in GLE in this study, data were insufficient to show sleep enhancement because they were present in such small amounts (3.76 and 3.90 µg/g) in the extract. Therefore, various polyphenols present in lettuce were analyzed using HPLC. As a result, chlorogenic acid, Q3G, and scutellarin, which are known to promote various physiological activities such as sleep and anxiolytic, sedative, and neuroprotective effects [38,39,40,41], were present at levels of 13.37 ± 0.24, 11.29 ± 0.39, and 2.57 ± 0.12 mg/g of extract, respectively (Figure 6).

The sleep-enhancing effects of chlorogenic acid, Q3G, and scutellarin, which are sleep-promoting active substances of GLE, were confirmed in a pentobarbital-induced sleep model. Chlorogenic acid, Q3G, and scutellarin all prolonged sleep time with increasing concentrations, and Q3G, in particular, reduced sleep latency (Figure 7). In addition, Q3G demonstrated that, similar to GLE, the sleep-promoting effect exhibits GABAergic action through competitive inhibition of 3H-flumazenil in a dose-dependent manner (Table 1). Therefore, it was revealed that the GABAergic interaction could be the main mechanism for the sleep-promoting effect of GLE, and Q3G acts as the main active substance. Quercetin and quercetin glycosides are abundant in traditional vegetables and are known to have a positive effect on neurocognitive protection [42,43]. In particular, quercetin extracted from vegetables can penetrate the blood–brain barrier and has the activity of inhibiting neuronal cell death by decomposing hydrogen peroxide [44,45]. Quercetin and Q3G act on GABA receptors, resulting in anxiolytic, sedative, and anticonvulsant effects [39]. In general, natural substances act on various neurotransmitter systems upon which sleep-related signaling molecules act. Neurotransmitters such as GABA, dopamine, and serotonin are also commonly used to treat mental illness [46]. GABA is a major inhibitory neurotransmitter in the brain; thus, GABA receptors may be a viable target for the anxiolytic or sedative effects of a natural herbal medicine. The GABA_A_ receptor is a hetero-pentameric ligand-gated ion channel responsible for fast inhibitory neurotransmission, and the α1β2γ2 receptor is its most abundant receptor subtype [47]. The α_2_-GABA_A_ receptor is expressed in hypothalamic and pontine nuclei. It has also been found that the α_2_-GABA_A_ receptor mediates anxiolytic effects of benzodiazepines such as diazepam and is thus a potential target for anxiolytic drugs with reduced hypnotic side effects [48]. Many insomnia therapies, including BDZ drugs, target GABA receptors. Several natural herbs and phytochemicals have also been shown to promote sleep through GABA receptors. For example, labdane diterpenoids of *Curcuma kwangsiensis* rhizome and phlorotannin of *Ecklonia cava* are known to act as positive GABA receptor modulators [49,50]. Polyphenolic compounds, including lutein, rutin, and Q3G, also act through the regulation of GABA receptors to exert anxiolytic effects in rats [40,51]. Our data demonstrate that the sleep-promoting effect of GLE is dependent on its interaction with GABA_A_ receptors (Figure 2 and Figure 5, and Table 1). However, since the current study did not address whether GLE or GLE-derived active chemicals interact with the GABA receptor in vivo, further research is needed.

## 5. Conclusions

In conclusion, GLE effectively improved sleep behavior in *Drosophila* and rat models in which physical stress was induced through GABAergic-mediated behavior. GLE has been shown to restore sleep patterns, relieving oxidative stress by removing ROS induced by physical stress. Among the various polyphenols contained in GLE, Q3G exhibited sleep-promoting activity and showed a sleep-promoting effect through interaction with GABA receptors along with GLE. This study provides evidence supporting the potential usefulness of the ethanol extract of green lettuce leaves in controlling oxidative stress-induced sleep disturbances.

## Figures and Tables

**Table 1:** Displacement (%) of [^3^H]-flumazenil binding of green lettuce leaf extract (GLE), chlorogenic acid, scutellarin, and quercetin-3-glucuronide (Q3G) through GABA_A_-benzodiazepine receptor assay.

Sample (Final Concentration, mg/mL)	0.1	1	10
Green lettuce leaf extract	1.36 ± 0.14	11.90 ± 1.35	16.17 ± 2.13
Chlorogenic acid	n.d.	n.d.	n.d.
Scutellarin	n.d.	n.d.	n.d.
Q3G	16.58 ± 0.39	51.11 ± 0.34	88.13 ± 0.70

Values are presented as the mean ± standard error of the mean (SEM) for each group, *n* = 3. n.d., not detected.

## Data Availability

The data that support the findings of this study are available from the corresponding author upon reasonable request.

## Supplementary Materials

The following are available online at https://www.mdpi.com/article/10.3390/antiox10060970/s1, Figure S1: Effects of GLE (green lettuce leaf extract) on survival rate by vibration stress in fruit flies.
