# Supplementary material for: Effects of Green Lettuce Leaf Extract on Sleep Disturbance Control in Oxidative Stress-Induced Invertebrate and Vertebrate Models
Source: Antioxidants (Basel). 2021 Jun 17;10(6):970. doi: 10.3390/antiox10060970 (PMC8234172; doi:10.3390/antiox10060970)
Supplement: Supplementary file 1 [file antioxidants-10-00970-s001.zip › antioxidants-1223712-supplementary.pdf]

Supplementary Materials

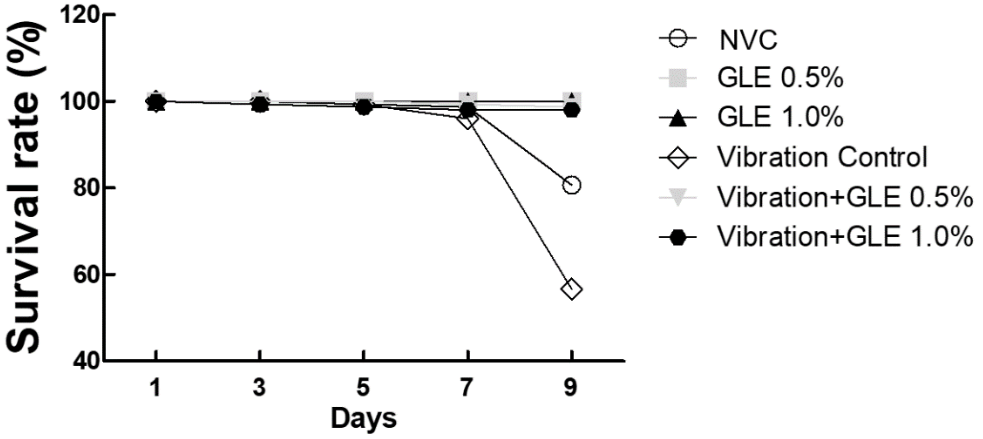

Figure S1. Effects of GLE (green lettuce leaf extract) on survival rate by vibration stress in fruit flies. Experimental groups include the no-vibration-stress control group (NVC), control group (stress-induced vibration), and GLE (green lettuce leaf extract)-treated groups with or without stress (0.5 and 1.0%).
